# Supplementary material for: Are carbon emissions trading and green financial instruments synergistic? -Comprehensive quantitative research based on content analysis
Source: PLoS One. 2024 Mar 7;19(3):e0298601. doi: 10.1371/journal.pone.0298601 (PMC10919601; doi:10.1371/journal.pone.0298601)
Supplement: S1 Data — (ZIP) [file pone.0298601.s001.zip › Data/地级市Z_F工作报告.zip!/╣π╢1⁄2/├╖╓▌╩╨/└┤╫╘╣1⁄2╓┌║┼í╛┬φ┐╦╩2╛▌═°í┐_2017.docx]

各位代表：

现在，我代表梅州市人民政府向大会作报告，请予审议，并请政协各位委员和其他列席人员提出意见。

过去五年的工作回顾

五年来，市政府在市委的正确领导下，在市人大和市政协的监督支持下，深入学习贯彻习近平总书记系列重要讲话精神，按照中央、省和市委的决策部署，抢抓“两大振兴政策”机遇，扭紧“三大抓手”，建设“一区两带”，全力加快振兴发展步伐，全市经济社会发展取得新进步。

一、综合实力不断提升。积极适应经济发展新常态，坚持稳中求进，推动经济行稳致远。2015年度粤东西北振兴发展评估考核，梅州排名第一。2016年，全市生产总值1045.56亿元、首次突破千亿元，固定资产投资650.36亿元，一般公共预算收入实现105.46亿元，分别比2011年增长54.9%、227.5%、124.9%，年均分别增长9.2%、26.8%、17.6%。工业加速发展，大力实施产业振兴三年行动计划，规划建设广东梅兴华丰产业集聚带，并上升为省级重大区域发展平台。全市新增省级园区4个， 9个产业园（集聚地）均享受省产业转移政策。2016年，全市规模以上工业增加值224.21亿元，年均增长10.3%；全市产业园区（集聚地）工业增加值比上年增长20.8%；技改投资完成75.87亿元，年均增长20.5%，总投资12.7亿元的“双喜·五叶神”专用生产线正式投产。县域经济实力明显增强，县域规模以上工业增加值实现157.85亿元，年均增长14.9%。深入实施乡贤回归投资兴业工程，五年累计引进亿元以上项目814个。现代服务业蓬勃发展，文化旅游、电子商务、现代金融等发展态势良好。2016年，第三产业比重超过44.2%，年均提升0.86个百分点；社会消费品零售总额619.77亿元、外贸进出口总额23.49亿美元，年均分别增长10.7%和11.5%。规划建设梅江韩江绿色健康文化旅游产业带，新增3A以上景区22个、四星以上酒店26家，建成20个海内外旅游推广中心，全市旅游接待人数、总收入年均增长27.9%和28.1%。新建10个电商产业园，快递网点实现镇（街）全覆盖。率先在全省建成金融生态市，新增2家上市企业和19家新三板挂牌企业，梅州客商银行成为粤东西北地区第一家获批筹建的民营银行。金融机构存、贷款余额分别比2011年增长1.9倍和2.1倍。特色高效农业成效明显，五年新建农民专业合作社3262家、富硒农业生产基地76个，新增省级农业龙头企业69家、总数居全省第一；粮食生产保持稳定，华南双季稻产量在兴宁创世界纪录；2016年，全市农业增加值211.89亿元，增长4.3%。质量强市扎实开展，产品、工程、环境质量主要指标提前达标。五年新增3个国家地理标志证明商标、6个国家地理标志保护产品。梅县获批全国客家文化生态旅游知名品牌创建示范区。

二、发展条件不断改善。以基础设施建设为重点，建成一批打基础、利长远、惠民生的重大项目。交通建设成效显著，区位环境得到提升，五年完成交通建设投资390亿元，年均增长22.4%。梅汕高铁全线动工；新增2条出省高速公路通道，实现县县通高速，通车里程新增223公里；五年累计升级改造国省道688公里、县乡公路1300公里；梅县机场新增8条航线，通航国内外15个城市，旅客吞吐量年均增长44.4%。梅州抽水蓄能电站开工建设，韩江高陂水利枢纽工程一期一段围堰合龙，粤电大埔电厂首期建成投产，我市成为粤东重要电力外送基地。完成1520公里山区中小河流治理和180公里梅江韩江沿岸重点堤围除险加固。中心城区扩容提质步伐加快，梅县撤县改区，嘉应新区起步区建设有序推进，江北老城区优化提升，建成一批产业项目和城市综合体，促进了产城融合发展。预计全市城镇化率达48.29%，比2011年提高5.02个百分点。全面开展人居环境综合整治，国家卫生城市通过复审，省卫生县城实现全覆盖。深入开展绿满梅州大行动，完成“一消灭三改造”任务，森林覆盖率达74.78%、居全省第二。成为广东唯一上榜2016中国“氧吧城市”前十名的城市。城区空气质量优良率达98.5%，饮用水源地水质达标率100%。全面完成省下达的节能减排任务，单位GDP能耗年均下降率为4.2%。大埔、丰顺调整纳入国家重点生态功能区。

三、社会事业不断进步。成功创建粤东西北首个教育强市。义务教育标准化学校覆盖率达99.1%；学前教育毛入园率、高中阶段毛入学率分别达95.15%、94%，比2011年提高7个、3.66个百分点；支持嘉应学院实施“创新强校”工程。启动创建卫生强市，县级公立医院改革覆盖所有县（市），基层医疗卫生机构综合改革基本完成。成立市中医医院。“单独两孩”和“全面两孩”政策顺利实施，计划生育服务管理改革有序推进。预防和减少出生缺陷工作成效显著，地中海贫血基因检测技术在全国推广。成功举办第三、四届世界客商大会，在海外设立15个经贸文化联络处。新增3处国家级、36处省级文物保护单位和11个省级非物质文化遗产项目，新建228个农村文化俱乐部和20个城区文化驿站。启动五华狮雄山建筑遗址保护开发省市共建项目。举办四届客家文博会，新建一批文化创意园区。振兴足球之乡十年规划取得阶段性成效，五华足球队成功冲甲。群众体育广泛开展，全民健身公共服务体系不断完善。关爱妇女发展和儿童成长，妇女儿童发展规划的实施通过省中期评估。国防教育和动员、兵役、双拥等工作扎实推进，两次成功创建全国双拥模范城市。外事侨务、港澳和对台工作有新进步，科技、人事编制、民族、宗教、统计、供销、气象、打私、应急、人防、档案、方志、残联和红十字会等工作取得新成效。

四、群众获得感不断增强。坚持每年办好十件民生实事，连续五年市级新增财力的80%以上用于民生事业。城乡居民收入持续增长，2016年全体常住居民人均可支配收入17986.6元，年均增长11.1%。积极扩大就业，五年城镇新增就业16.05万人、劳动力转移就业43万人次，培训劳动力19.9万人，扶持创业1.8万人；城镇登记失业率保持在2.45%以内。建立完善社保制度体系，基本实现人人享有社会保障的目标，基本社会保险覆盖率保持在95%以上；底线民生项目补助标准逐年提高，社会救助标准动态调整机制不断健全；社会养老服务体系初步建立，每千名老人拥有养老床位27.9张。全面完成保障房建设和棚户区改造任务。新农村建设有新成效，3个省级新农村连片示范建设扎实推进，8个原中央苏区县幸福村居示范片（线）建设基本完成；培育省级名镇名村59个、省宜居示范城镇11个、示范村庄14个，46个村列入中国传统村落名录、居全省首位。农村超高速无线局域网应用试点加快推进，光纤接入用户超过16万户；农村电网改造升级惠及20.8万用户；实施农村饮水安全工程，解决了130多万群众饮水安全问题。农村生活垃圾有效处理率达75.41%。乡村金融服务站、助农取款服务点实现全覆盖。建成广东首个林业产权流转交易服务平台。圆满完成两轮扶贫“双到”工作，937个村8.1万贫困户32.5万贫困人口实现了稳定脱贫。

五、发展内生动力不断激活。进一步优化投资发展环境，创新创业氛围日益浓厚。坚持先行先试，以点带面，推进26项中央、省部署的改革试点，实施252项“微改革、微创新”项目，公布市、县权责清单，市级行政审批事项压减53.7%、审批时限缩短64%。供给侧结构性改革五个专项计划协同推进，“去降补”取得了阶段性成效。商事制度、投融资体制等改革全面推进，五年新增各类市场主体10.5万户、年均增长11.5%。自觉接受监督，坚持向市人大及其常委会报告工作，向人民政协通报情况，建议提案按时办结率、代表委员满意和基本满意率达100%。严格落实中央八项规定，深入开展党的群众路线教育实践活动、“三严三实”专题教育和“两学一做”学习教育，工作作风进一步转变。加强法治政府建设，完善市政府工作规则，建立法律顾问制度，行政复议案件受理率、结案率均达100%。完成“六五”普法任务。加强正风肃纪，廉洁政府建设取得新成效。深入推进政务公开和廉政监管，行政服务大厅、网上办事大厅和"12345"政府服务热线建成运行。落实行政效能投诉办理责任制，强化重点领域审计监督，政府公共服务总体满意度连续三年居粤东西北首位。创新社会治理，首次获评全国创新社会治理优秀城市，群众安全感和政法工作群众满意度连续5年全省第一。广东省雁洋公益基金会成为首家省级非公募基金会。依法规范信访秩序，强化安全生产、食品药品安全监管，社会保持和谐稳定。

各位代表，五年来，梅州经历了发展的艰辛，战胜了各种困难和挑战，取得了来之不易的成绩。在这里，我代表市政府，向全市人民，向中央和省属驻梅单位、驻梅部队，向所有关心支持梅州发展的海外侨胞、港澳台同胞和社会各界人士，表示衷心的感谢并致以崇高的敬意！

五年来的成就，为今后的发展打下了坚实的基础；五年来的实践，为继续前进积累了宝贵的经验。我们更加深刻体会到：只有紧密团结在以习近平同志为核心的党中央周围，沿着正确的方向前进，始终把抓发展、促发展作为我们的工作重心，才能早日实现振兴发展的目标。只有紧密结合梅州实际，扬长补短，始终把重实体、兴实业作为我们的重要抓手，支撑经济跨越发展，才能加快振兴发展的步伐。只有坚持务实重干的工作态度，勤政廉洁、攻坚克难、勇于担当、善作善成，才能凝聚全市上下团结奋进的强大合力。只有察民情、顺民意，竭尽全力为群众办好事、实事，真正做到惠民、利民、为民，才能赢得群众的信任和支持。

在肯定成绩、总结经验的同时，我们也清醒地看到发展中存在的困难和不足，主要是：经济总量还不大，与山区人口大市的地位不相称；实体经济薄弱，产业集聚度低，尤其是工业发展短板非常突出，创新创业氛围不够浓厚；交通、土地、资金、人才和环境容量等制约因素仍然存在；中心城区辐射带动能力弱，城市的功能和承载力亟待提升；公共资源配置不尽合理，在入学、就医、养老等方面与群众的需求仍有较大差距，等等。这些困难和问题，是今后工作的努力方向，我们将高度重视，切实加以解决。

今后五年的目标任务

各位代表！市第七次党代会绘就了梅州今后五年发展的宏伟蓝图，提出加快构建“一区两带六组团”发展新格局，是对梅州产业发展、区域发展的新谋划，是梅州振兴发展路径的新拓展，为今后五年的政府工作指明了方向。从当前的发展趋势判断，影响全球发展的不确定、不稳定因素仍然存在，国内经济下行压力仍然较大。但是我们也要看到，随着供给侧结构性改革的成效逐步显现，经济持续增长的有利条件和积极因素正在集聚；随着创新驱动发展战略的深入实施，新技术、新产业、新业态层出不穷，互联网经济、分享经济方兴未艾，为我们站在新的起点谋划产业发展，推动传统产业转型，培育经济发展新动能带来新的机遇和条件，也为我市弯道取直、后发赶超，提供了很大的发展空间。省委、省政府全力推进珠三角与粤东西北一体化发展，释放了政策利好的新信号；穗梅产业共建迎来“窗口期”，未来两三年内将落地一批优质项目，加速经济发展。特别是，随着“两大振兴政策”的深入实施，我市的交通条件、区位因素逐步发生改变，产业集聚平台效应日益显现，城市整体功能和辐射带动作用不断增强，支撑梅州振兴发展的条件更加具备。未来五年，是梅州大有可为的时期。只要我们因地制宜，遵循和把握发展规律，坚定发展信心，凝共识聚合力，汇众智谋发展，就能够把宏伟蓝图变成美好现实。

今后五年，政府工作的总体要求和目标任务是：深入贯彻习近平总书记系列重要讲话精神和治国理政新理念新思想新战略，积极践行“五大发展”理念，坚持稳中求进工作总基调，围绕振兴发展目标，按照市第七次党代会的部署要求，紧扭“三大抓手”，坚定不移发展新型工业，大力发展绿色健康文化旅游特色产业，致力改善基础设施条件，推动中心城区与县域组团发展，守住发展底线，做大经济总量，提升民生福祉，加快构建“一区两带六组团”发展新格局。至2018年，全市生产总值和城乡居民人均收入力争比2010年翻一番，全市农村相对贫困人口全部脱贫，与全省同步全面建成小康社会；至2021年，全市生产总值力争突破1600亿元、年均增长8.5%左右，人均生产总值达到3.7万元、年均增长8%左右。未来五年的政府工作，重点在以下五个方面谋求突破：

一、突出产业集群建设。经济发展的根本动力在产业，我们必须致力补齐产业发展短板，推动产业集聚升级。围绕产业共建，全力推进广东梅兴华丰产业集聚带建设，打造汽车零配件、新能源新材料及先进制造业等产业集群。坚持创新驱动发展，推动传统产业转型升级，培育发展一批战略性新兴产业，打造绿色产业发展基地。争取通过五年努力，培育形成3个以上产值超百亿的产业群。推动梅江韩江绿色健康文化旅游产业带上升为省级重大区域发展平台，以全域旅游带动文化、旅游等产业向高端发展，创建成为国家旅游休闲示范城市、国家全域旅游示范区，实现区域内旅游产业增加值年均增长13%。

二、突出中心城区和县域经济发展。城市既是经济发展载体，更是人们的生活家园。围绕建成粤闽赣边区域性中心城市，以中心城区扩容提质为龙头，以六个县城建设为支撑，以生态修复、城市修补为抓手，提升城市形象和品位。转变城市发展方式，完善城市功能，吸引人口进入，探索发展梅州置业、周边地区工作的“双城生活”模式。按照“六组团”的功能发展定位，以“一县一特色”组团发展县域经济，促进城乡一体化发展。至2021年，中心城区起步区基本建成，全市城镇化率达55%，常住人口达到70万人左右，县域生产总值突破千亿元。

三、突出软硬环境建设。良好的投资发展环境，是提升区域综合竞争力的重点所在。我们将致力建设内联外通的综合交通运输体系，五年内新建成6条总长265公里高速公路，提升干线公路等级，优化路网布局，推进机场迁建立项，确保2019年迈入“高铁时代”，打造珠三角、汕潮揭东进北拓内陆腹地的桥头堡。完善水利、能源、信息等基础设施建设，改善振兴发展条件。围绕创建国家森林城市、国家环保模范城市，深入开展城乡环境综合整治和美丽乡村建设，让天更蓝、水更绿、城乡更宜居。全面推进依法治市。加大简政放权力度，提高政府执行力，建设新时期服务型政府。

四、突出改革和创新。深化改革创新，激发市场活力和社会创造力，是推动我市加快振兴发展的强大动能。我们将大力推进供给侧结构性改革，统筹抓好行政审批、商事制度和医药卫生、文化教育、监察体制等各领域改革，引领经济发展。强化科技创新，充分发挥院士团队等智库作用，以省市共建专利技术孵化产业园、人才孵化基地等创新平台为抓手，扎实推进众创空间和专业镇建设，促进大众创业、万众创新。

五、突出社会民生事业改善。全面建成小康社会，需要大力发展民生社会事业，提供更多的优质公共服务。围绕建设“广东文化高地”，传承和弘扬客家优秀文化，繁荣发展文化事业。推动教育优先发展，确保2018年成功创建“广东省推进教育现代化先进市”。推进省市共建，支持嘉应学院办成国内知名特色大学。实施健康梅州工程，支持市人民医院建成粤闽赣边区域性医疗中心、市中医医院等创建三甲医院，提升基层医疗卫生服务能力，基本建成卫生强市。发展体育事业，振兴足球之乡。坚持民生为重，每年办好十件民生实事。坚持因人施策、精准施策，确保349个省定相对贫困村和63045户175917名相对贫困群众如期实现精准脱贫。

2017年工作安排

五年宏图，重在开局。做好今年的政府工作，必须保持战略定力和工作韧劲，坚持稳中求进，把供给侧结构性改革作为经济发展主线，全面做好稳增长、促改革、调结构、惠民生、防风险各项工作，努力开创梅州经济社会发展新局面。

今年的主要预期目标是：全市生产总值增长8.0%左右；固定资产投资增长20%，社会消费品零售总额增长10%，进出口总额增长5%；一般公共预算收入增长10%；居民消费价格指数控制在3%以内，居民人均可支配收入增速与经济增长基本同步；城镇登记失业率控制在3.5%以内；节能减排降碳约束性指标完成省下达年度计划。

实现今年的目标任务，重点是抓好以下九个方面工作：

一、坚定不移发展以工业为核心的实体经济，推动经济扩量提质

重实体、兴实业，既是全市上下的一致共识，也是立市之本、强市之基、富民之源。

加快建好重大产业平台。统筹推进广东梅兴华丰产业集聚带建设，以广梅园建设为重点，同步推进周边县（市、区）工业园区建设，打造工业发展新引擎。坚持特色发展、错位发展，广梅园重点发展高新技术产业和先进制造业，梅江东升园重点发展电子信息产业，梅县水车园重点发展生物医药大健康产业，兴宁水口园重点发展机电、机械制造和食品饮料产业，五华河东园重点发展先进装备制造、自行车产业，丰顺埔寨园重点发展电声、医药产业，蕉华工业园重点发展食品医药、新型建材产业。优先完善基础设施，重点推进园区交通、能源、通信、环保等配套设施建设，争取年底前完成梅畲快线主体工程。积极争取和用好省专项扶持资金，大力推进污水处理、固体废物处置和供排水等水务环保设施项目建设。抓紧规划建设广梅医院、职业技术学校和商务中心，完善生活配套。积聚创新发展要素，加快推进创新创业孵化园、松棚铁路货场和深圳有信达保税物流等重点项目建设，争取尽快获批综合保税区。调整完善土地利用总体规划，抓好土地征收、储备工作，确保核心区新增4000亩建设用地。

全力推动产业共建。全面落实省扶持产业共建政策，建立广梅园畲江片区协调联席会议制度，探索推进总部、研发和孵化环节在广州，配套和生产环节在梅州的共建模式，力促产业集聚区加快建设。发挥广汽集团的龙头作用，年内新增3个动工项目，打造广汽汽车零部件产业园。以广药集团为核心，带动周边配套企业进驻，推动形成广药王老吉大健康产业园。主动对接广州开发区，加快推进新能源新材料及先进制造产业园、食品产业园规划建设。充分发挥我市的主体作用，强化与广州市的沟通协调，全力抓好征地拆迁、土地整理、道路建设等前期工作，确保项目无障碍建设、早见成效。

大力发展民营经济。鼓励全民创业，培育壮大市场主体，力促中小微企业遍地开花。积极推动“个转企”、“小升规”，新增65家民企上规模，推动300家以上企业进入“重点中小微企业池”。借力资本市场，鼓励支持上市公司实行兼并合作、增资扩股、技术合作等方式，做大堆头。扶持有资质的企业上市，力争今年新增4家以上新三板挂牌企业。加强政银企合作，整合资源组建市级投资开发公司，积极发展融资租赁公司、小额贷款公司和融资性担保机构，争取新增贷款150亿元，保障优势产业、重点项目融资需求。

推动企业技改升级。实施创新驱动战略，全面落实《中国制造2025》和省实施意见，用好省技改政策和资金，鼓励企业增资扩产、技改升级、设备更新，推动机器人及智能设备应用。抓好塔牌600万吨旋窑水泥生产线等重点技改项目，推动烟草、水泥、电力、陶瓷等传统支柱产业转型升级。深化产学研合作，在企业和高校、科研机构之间搭建平台，共建研发中心和创新服务中心，引导创新要素向企业集聚。

创新开展招商引资。坚持招商项目数量与质量并重，只要不触及环保底线、生态容量合适，就多多益善。认真研究珠三角、汕潮揭等地区的产业发展动向、产业转移规律，围绕产业的前延后伸和主导产品及其上下游产品，开展链式招商、以商引商，争取引进上下游关联配套项目，加快培育标杆企业、龙头企业。围绕工业园区产业布局，突出重点产业对口招商，提高招商精准度。进一步拓展招商网络，选聘招商顾问，开展中介招商。落实各级招商责任，设立产业扶持基金，完善外出乡贤信息库，争取更多乡贤回乡共襄发展。

二、大力推进交通等重点基础设施建设，发挥投资拉动的重要作用

对经济欠发达的梅州而言，投资和项目拉动仍然是当前推动经济增长的主要动力。

建设快速综合交通体系。强化互联互通，大力实施一批交通基础设施项目。加快推进梅平、大潮（含大漳支线）、梅州东环高速建设，确保无障碍施工；全线建设大丰华高速丰顺至五华段、兴汕高速五华至陆河段；确保兴华高速（含畲江支线）建成通车。加快推进梅汕高铁建设，力争年内动工建设双龙、瑞梅铁路。抓好梅县机场迁建前期工作，规划建设五华通用航空机场。大力推进国省道和县乡道路的升级改造，动工建设300公里国省道改造工程。争取立项建设国道G205线、G206线梅州城区改线工程。抓好航道整治，改善通航条件。

推进水利能源等重点项目建设。积极创造条件，着力推进梅州抽水蓄能电站征地、移民等工作，确保9月份主体工程如期开工。加快建设韩江高陂水利枢纽工程，确保完成年度投资任务。大力推进中小河流综合治理，完成170公里年度整治目标。争取动工建设益塘水库引水工程。完善城乡生活污水处理基础设施。推动新一轮农村电网改造升级，实现动力电入村。

加快建设信息基础设施。加大与各通信运营商合作，推进光纤入户，抓好原中央苏区农村超高速无线局域网建设和应用，全面提高城乡宽带覆盖范围。动工建设飞翔云计算等智慧城市项目。通过购买服务，实现城区公园等公共场所WIFI全覆盖。规划建设“粤闽赣+”应急云示范基地，加快构建政务服务大数据库，促进信息化和工业化、农业现代化、新型城镇化融合发展。

强化项目保障。创造性解决项目建设中的资金、用地、环评等问题，力促年度总投资565亿元的250个重点项目落地，提高投资质量和效益。深化对原中央苏区等政策的研究对接，瞄准国家、省产业战略和投资方向，争取中央、大型国企倾斜性投资，切实把政策红利转化为发展红利。加强项目库建设，抓好项目筛选、论证和申报等前期工作，储备一批打基础、利长远的项目。优化投资结构，鼓励引导民间资本参与市政、社会事业等重点项目建设。

三、加快中心城区扩容提质，致力建设宜居城市

推进以人为核心的新型城镇化，回归城市本源，是城市工作的目标指向。

高起点规划城市。进一步完善城乡规划体系，编制《梅州市新型城镇化规划》，争取尽快获批《梅州市城市总体规划（2015-2030）》，引领城市发展。坚持“多规合一”，融入“双修”规划理念，争取年内完成市政道路、供排水、亮化美化等专项规划，年底前基本实现城市近期建设规划范围内控规全覆盖。优化城乡空间布局，统筹规划建设梅州中心城区、兴宁副中心城市和五个县城。推进规划行政许可制度改革，争取年内理顺市与梅江区、梅县区的规划管理体制，促进规划统一、管理科学。

高标准建设城市。树立城市是幸福家园的理念，按照海绵城市、综合管廊城市试点标准，加快推进嘉应新区起步区建设，完善城区排水防涝、污水处理等基础设施和教育、医疗等公共服务供给，年底前基本完成江南新城、芹洋半岛、梅县新城的主要道路管廊和安置区主体建设。促进城市互通，确保上半年建成客都大桥、完成罗乐大桥主体工程。规划建设梅塘湾休闲健身公园。加快推进剑英公园改造等公共服务配套建设，年底前基本建成城市馆、青少年科技馆、地下停车场等项目。注重产城融合，加快推进产业项目建设，推动城市发展。实施“双修”工程，推进城区周边山体和梅江河、周溪河的生态修复，对城市形态、建筑色彩风貌、广告牌匾、绿化景观、夜景亮化等进行城市修补，提升城市形象。发挥梅江区的主体作用，加快推进教育文化新区、保健疗养社区规划建设，改造江北老城区街头老屋等公共绿地景观，提升人居环境。

高效能管理城市。创新城市治理方式，探索建立城市管理委员会，理顺市和区城市管理执法体制，科学下放职权、人员编制，提升城市管理精细化水平。整合平安梅州、数字城管等资源，打造城市综合管理信息平台和视频监控系统，加快智能配电网建设步伐，建设智慧城市。实施市政道路顺畅工程，加大“治堵”、“治超”力度，综合治理城区道路交通秩序。编制城区现状道路慢行系统规划，加快建设外环路、学院路至国道G205道路、客都大桥东端连接线，改造城区华南大道与梅塘东路交叉口。探索引进社会资本共建立体智能停车场，逐步解决停车难问题。开展城管服务进社区，提高市民素质，实现城市共治共管、共建共享。

高效益整合资源要素。有针对性地解决城市建设过程中面临的土地、资金等难题。规范并加强土地管理，优化国土空间开发，用好“三旧”改造、棚户区改造等政策，加快处置批而未供和闲置土地，进一步盘活存量、做优增量，提高土地投资强度和单位产出值。创新投融资机制，积极争取中央和省专项资金支持，加强与政策性银行合作，设立梅州市交银原中央苏区振兴发展扶贫基金，大力推广PPP、政府购买服务、政府债券等融资方式，提高重大项目股权、债权直接融资比重，实现城市建设投融资平衡。

四、培育发展新兴产业，打造新的经济增长点

坚持多元驱动，加快发展以现代服务业为重点的第三产业，对支撑我市经济发展具有现实的意义。

大力发展全域旅游。明晰市场定位，彰显发展特色，加快建设梅江韩江绿色健康文化旅游产业带。以创建“国家全域旅游示范区”、“国家旅游休闲示范城市”为抓手，完善公共停车场、旅游厕所、游客服务中心等配套设施，推进重点景区提质升级。适应大众旅游时代，科学设计旅游线路，积极打造“一县一品”、“一镇一特”、“一村（景）一型”，促进旅游业全区域、全要素、全产业链发展。实施“旅游+”模式，建设旅游大数据中心，推动旅游与相关产业融合发展，不断丰富旅游产品供给。创新旅游经营模式，推行市民旅游一卡通，依托“一带一路”城市旅游联盟、红色旅游联盟等平台，加大旅游营销推广力度，扩大梅州旅游影响力和吸引力。

推进“互联网+”深入开展。大力推动“互联网+”与社会生产生活的深度融合。围绕创建国家电子商务示范市，出台相关扶持政策，加快电子商务进农村综合示范县建设，推动邮政、快递服务向农村延伸。主动适应消费市场需求，建设跨境电商分拣清关中心。引导经营服务向“互联网+”转变，扩大便利消费。依托海吉星农产品商贸物流园、华泰农产品交易中心、中国供销·粤东农批等平台，大力发展农产品仓储、冷链物流、第三方物流等产业形态，优化升级专业市场。开展电商招商，吸引知名电商平台、企业和中介服务机构入驻。

发展先进服务业。顺应市场需求，推动生产性服务业向专业化和价值链高端延伸、生活性服务业向精细化和高品质转变。加快发展金融保险、服务外包、信息技术、检验检测等生产性服务业，推进服务业与一二产业协调发展。擦亮长寿乡金字招牌，推进医养结合，发展养老服务。大力发展创意经济，培育和引进研发机构、文化创意等高端服务业，以文化创意的理念推动陶瓷、电声等产业发展。设立文化产业基金，建设文创研发、展示、交易平台，高标准建设文化产业园区，加快打造文化产业“梅州板块”。完善体育综合体经营管理，重点发展健身休闲、竞赛表演、场馆服务等体育服务业。拓展信息、住房、家政等消费领域，努力满足人们多层次、多元化消费需求。

五、统筹城乡一体化，推动县域发展上新水平

小河有水大河满。提升市域经济整体实力，需要县县联动、城乡互动，走组团式、特色化发展的路子。

改善县域发展条件。落实县域经济社会发展扶持政策，着力改善交通、电力、水利、环保、信息等基础设施，加快公共服务向农村覆盖。抓好县、乡、村公路升级改造和管护，畅通县域交通。以县城为中心，加快推进新区建设，完善教育、医疗、文化、体育等功能布局，提升县城辐射带动能力。优化城镇体系空间布局，促进农村人口向中心镇有序流动，全市22个中心镇年内均规划建设一个住宅小区、商业街和政务中心。深化农村综合改革，全面开展农村土地承包经营权确权登记颁证工作，分类推进农村集体资产确权和股份制改革，激活农村发展活力。

抓好县域产业发展。依托各县（市）产业基础和资源禀赋，加大招商引资力度，重点培育2-3个特色产业，带动上下游企业协调配套发展，打造特色支柱产业集群。创新管理体制和运行机制，支持各县（市）设立园区投资开发公司，推动工业进园区，集聚发展、做大做强。因地制宜培育发展农业观光、休闲度假和健康养老等特色产业，提高专业镇发展层次。借力穗梅区县结对帮扶，推动一批重点项目落地。强化融资、用地、用工、用电等要素支撑，提升县域产业转移承接能力。

推进农业现代化。坚持以工业化的理念经营农业，促进农业向高质量、标准化、集约化方向迈进。围绕发展有市场竞争力、高附加值、知名品牌的高效农业，坚持科技兴农，优化供给结构，更好地引导农业生产。打响富硒长寿品牌，扎实推进“一镇一品”、“多村一品”建设。开发精深加工农副产品，加快发展“互联网+农业”，发展壮大农业龙头企业，拓宽农产品销售渠道，提高金柚、脐橙、茶叶、油茶、优质稻米和水产、南药等绿色产品效益。发展社会化服务组织，培育新型经营主体和新型农民。发挥绿色资源优势，大力发展生态农业和森林旅游，培育林下经济。严守耕地红线，严处乱占耕地等行为。扎实推进第三次全国农业普查工作。稳定粮食生产，确保粮食安全。

建设特色镇和美丽乡村。抓紧编制《县（市）域乡村建设规划》，引领新型城镇化和新农村建设。学习借鉴江浙地区发展特色小镇的做法，突出地域和历史文化特色，以点带面，支持梅县松口、丰顺公式隍、大埔百侯等镇向特色化、产业化发展。大力推进农村人居环境综合整治，每年整治20%以上的自然村。进一步规范农村建房，加快培育省级名镇名村，发展乡村游、民俗游等，打造宜居宜游的美丽乡村。引导金融和社会资本参与农村建设，推动城乡基础设施和公共服务设施互联互通、共建共享。

扎实开展精准扶贫、精准脱贫。提高贫困人口的自我发展能力，注重因户因人精准施策，灵活采取创业、就业、教育等帮扶措施，突出产业扶贫，多渠道增加贫困户收入。重视抓好困难家庭和残疾人家庭子女的教育帮扶，阻断贫困的代际传递。健全脱贫长效机制，完善低保、社保、教育和残疾人等数据台账，建立农村低收入群体大数据库，做到精准到人、长期帮扶。整合各类涉农资金和帮扶资金，推进贫困村基础设施建设，改变贫困村落后面貌。广泛发动社会各界参与，通过扶贫济困、金融贷款和众筹、众包等方式，形成全社会支持参与扶贫开发的良好格局，确保今年全市70%以上相对贫困人口实现脱贫。

六、坚守发展底线，提升城乡环境质量

保持定力，守护好梅州的绿水青山，是我们的责任担当。

巩固生态文明成果。深入推进国家生态文明先行示范区建设，严格划定生态红线，注重“一区两带六组团”核心地段生态修复，筑牢生态安全屏障。以创建国家森林城市为契机，深入开展新一轮“绿满梅州”大行动，加强林业重点生态工程建设，新建森林公园16个、湿地公园3个。加强森林资源的抚育和管护，实施《梅州市森林火源管理条例》，对森林火灾“零容忍”。完成国有林场改革。落实最严格水资源管理制度，深入开展南粤水更清行动，加强饮用水源水质保护，保障水安全。

推进绿色低碳发展。坚持绿色发展理念，推动生产、流通、消费各环节的循环经济发展。狠抓节能减排，推进节能减排财政政策综合示范城市建设，实施清洁生产、环保基础设施、节能技术改造等项目，完成黄标车、高污染燃料锅炉淘汰任务。大力发展环保产业，培育发展环保能源发电、风电等企业。推广应用新能源汽车。积极开发森林碳汇项目。推广节能环保建材应用，提高建筑节能标准。严格落实项目准入条件，严把环境准入关。扎实推进节能型机关建设，增强全民环保意识。

加强城乡环境综合整治。继续推进创建国家环保模范城市各项工作。严格落实城乡环境综合整治测评办法，加大问责力度，扎实开展城乡“脏乱差”、“两违”和城市内涝、大气、水、土壤污染等综合整治。深入开展爱国卫生运动。严格实行市政府挂牌督办重点环境问题制度，推动垃圾处理、污水处理、供水供气等项目建设。保持城区全年空气质量优良。

七、深化改革和扩大开放，进一步激发发展活力

改革开放是梅州加快振兴发展的永恒主题。我们要以改革促攻关，以开放促跨越。

推进重点领域改革。切实把经济重心转到供给侧，适度扩大总需求，扩大有效供给和优质供给。落实“去降补”措施，完成补短板年度324项706.61 亿元投资任务。推进国有“僵尸企业”出清重组，落实国家和省“零收费”、“营改增”等政策，促进企业降本增效。深化放管服改革，加快政府职能转变。继续削减工商登记前置审批事项，同步取消后置审批事项。开展“减证便民”行动，推广电子营业执照和“同城通办”，探索“证照分离”改革试点。实施“互联网+政务服务”模式，推进一门式一网式行政服务平台建设。深化投融资体制改革，完善政府投资管理办法。

提高对外开放水平。坚持“走出去”，加强与“一带一路”沿线国家和东南亚客属地区的交流合作，开辟新的市场和商机。抓好对外贸易，争取1个以上省级经济开发区列入省加工贸易梯度转移重点承接地，培育一批特色产业申报省级外贸转型升级基地。加大力度推广大埔陶瓷、丰顺电声等区域品牌，推动机电、家具等特色产业走向国际市场。发挥梅州青蒿素药品援非抗疟的作用，加强与非洲国家医疗卫生合作。深化海峡两岸交流合作，推动梅港澳台青少年交流合作品牌化、基地化、项目化建设。积极对接汕潮揭、赣南和闽西地区，推进要素自由流动、资源高效配置、市场深度融合。办好第五届世界客商大会。

推动创新创业。充分发挥我市创新创业“三大资金、两大平台”的作用，办好创新创业大赛，推广应用创业券创新券，培育发展一批创新企业、创业团队。大力推进专利技术孵化产业园建设，年内建成1家省级科技企业孵化器，推动众创空间县（市、区）全覆盖。实施更开放的创新人才引进政策，推进院士创新创业驿站、新型智库和人才孵化基地等平台建设，吸引高端人才集聚。推进质量强市建设，培育一批驰名商标、著名商标、名牌产品和国家地理标志证明商标、保护产品。完成国家水泥及制品质量监督检验中心、广东省质量监督电声产品检验站（梅州）建设。

八、重视社会民生建设，让全民共享发展成果

坚持共享发展，提升人民群众更高层次的获得感和幸福感，是政府工作的职责所在。

推进教育现代化。巩固提升“教育创强”和“全国义务教育发展基本均衡县”成果，加快创建“广东省推进教育现代化先进市”。统筹推进城乡义务教育一体化发展，科学布局中小学校、幼儿园。办好东山中学、曾宪梓中学等10所示范性高中。坚持面向市场、服务发展、促进就业，加快推进梅州南方紫琳职业学院建设，培养具有工匠精神的专业技能人才。推进嘉应学院省市共建，改善办学条件，支持创新发展。拓展社区教育，提升开放教育水平。实施“名师工程”，塑造提升一批名校长、名教师。实施教育大数据应用工程，加快梅州智慧教育项目建设。

发展文化体育等各项事业。依托客家文化、红色文化等资源，以争创全国文明城市为龙头，推进城乡四级联创，带动“四城”同创，加快建设文明客都。围绕建设“广东文化高地”，落实文化强市规划纲要，推进现代公共文化服务体系建设。保护和活化利用历史文化遗产、红色遗迹，抓好中国客家博物馆、非遗馆等展陈升级，推进五华狮雄山建筑遗址公园建设，推动围龙屋申报世界文化遗产工作。繁荣客家文化，打造一批文艺（戏曲）精品。落实振兴足球之乡规划，支持职业足球发展，推进足球文化公园建设。积极承办各类体育赛事，扩大城市知名度。开展南粤古驿道定向大赛，打造全民健身特色品牌。办好第八届市运会。加强国防动员工作，支持军分区和人武部建设。进一步做好国家安全、人防、双拥、对台、妇女儿童、气象、防灾减灾、民族、宗教、统计、方志、档案、打私、打假、应急等各项工作。

完善医疗和社会保障体系。推进卫生强市建设，探索发展医联体模式，构建利益、责任共同体，方便百姓就医。推进家庭医生签约服务，落实基层首诊、分级诊疗。加快市中医医院改造升级、市第三人民医院整体迁建步伐，支持广州中医药大学紫合梅州医院、东山医院等民营医院加快建设。推进各县（市、区）危重孕产妇和新生儿救治中心建设，提升妇幼保健服务能力。启动城市公立医院综合改革。严格落实疾病防控措施，提升突发公共卫生事件应急处置能力。实施全民参保登记计划，推动社保扩面征缴。推进医疗保险城乡一体化和机关事业单位养老保险参保工作，提高低保、五保标准和医疗救助水平。以丰顺县为试点，推动各县（市、区）创建广东省食品安全示范县，保障食品（含食用农产品）安全。发展社会慈善事业，更加关爱贫困、病残、老年妇女、单亲母亲和孤儿、留守儿童等特殊困难群体，让他们拥有更多的幸福感。

创新社会治理。建立鼓励基层排查化解矛盾纠纷考核机制，提高预测预警预防各类风险能力。积极运用大数据技术推进社会管理创新，构建公众参与、社会协同的治理模式。健全律师服务基层治理长效机制，建立覆盖全市的法律援助服务圈。推进“七五”普法，加强信访法治化建设。强化网络舆情监督引导，拓宽网上信访反映渠道。健全安全生产监管责任体系，对重大隐患、安全风险实施分级挂牌督办、跟踪监管。抓好禁毒工作，提升全民识毒拒毒能力。加强社会治安防控体系建设，完善镇街综治信访维稳中心功能，打造“两所三站四纳入”新型综合实战平台，新建760个城区视频监控点，不断提高社会治理社会化、法治化、智能化、专业化水平，建设更高水平的“平安梅州”。

办好十件民生实事。一是提高底线民生保障水平。城乡居民基本养老保险基础养老金标准从每人每月110元提高到120元。城镇、农村低保人均补差水平分别从每月419元、191元提高到457元、206元。农村特困人员救助供养平均标准提高到每人每年8088元以上，不低于所在县（市、区）上年度农村居民人均可支配收入的60%。孤儿基本生活最低养育标准集中供养水平从每人每月1340元提高到1450元、分散供养水平从每人每月820元提高到880元。城乡医疗救助住院补助平均标准从每人每年2178元提高到2828元，城乡低保对象基本医疗救助比例达到80%以上。二是加大扶贫济困力度。建档立卡贫困户子女每人每学年生活费补助标准提高到小学和初中3000元、高中和中职（含技工）5000元（含国家助学金）、高等专科10000元（含国家助学金），免学费补助标准提高到高中2500元、中职（含技工）3500元、高等专科5000元。新开工建设3791套（户）棚户区改造安置房，基本建成2809套（户）；新增发放租赁补贴250户；完成农村危房改造9180户。三是关爱特殊群体。困难残疾人生活补贴从每人每年1200元提高到1800元，重度残疾人护理补贴从每人每年1800元提高到2400元；新建53个、规范运行58个社区残疾人康园中心。新建艾滋病检测确证实验室1间、检测筛查实验室10间、检测点100个。支持社区矫正中心规范化建设，对全市社区服刑人员实行教育矫正。四是提升医疗卫生保障水平。城乡居民基本医疗保险补助标准从年人均420元提高到不低于450元。职工医疗保险基金年度累计最高支付限额从8万元提高到9万元，城乡居民基本医疗保险基金年度累计最高支付限额从45万元提高到50万元。职工医疗保险门诊特定病种报销比例统一调整为75%，城乡居民医疗保险门诊特定病种报销比例从60%提高到70%，门诊特定病种年度支付限额按病种在原限额基础上提高1000元至6.5万元不等。城乡居民医疗保险普通门诊每人每日每次最高支付限额从30元提高到100元，年度累计最高支付限额从150元提高到500元，报销比例从50%提高到60%。城乡居民大病保险起付标准从1.2万元降为1万元，报销比例统一调整为70%，年度最高支付限额从15万元提高到16万元；城乡五保供养对象、建档立卡贫困人员的大病保险起付标准下调至2000元，报销比例提高到80%，不设年度最高支付限额。规划扩建梅州市妇幼保健计划生育服务中心。升级改造全市2040间村卫生站。五是促进城乡义务教育一体化发展。义务教育寄宿制公办学校公用经费补助从农村扩大到城市，补助标准从每生每年200元提高到250元。对城乡义务教育家庭经济困难寄宿生，按小学每生每年1000元、初中每生每年1250元标准给予生活费补助。农村边远学校教师生活补助标准提高到每人每月不低于900元。六是完善城区公共服务设施。改造城区670个小区供水管网。动工建设城区2所学校和2个公共停车场。建设华南大道田家炳医院侧、彬芳大道喜多多超市侧、嘉应中路学艺路口3个地段人行天桥和锭子桥至嘉应学院西校门路段16座公交站亭。建设新峰桥至205国道30米路。完成100个“文化驿站（台）”建设。七是促进就业和创业。扶持创业2500人、城镇就业25000人、失业人员再就业23000人。补贴参加技能晋升培训的劳动者1万人。八是改善群众生产生活条件。实施266宗村村通自来水工程建设。完成400公里农村公路路面硬化改造。在全市农贸市场开展食用农产品快速检测。城镇独生子女父母计划生育奖励标准从每人每月80元提高到120元。九是加强环境治理。建设34座污水处理厂、330公里污水管网和1580座农村污水处理设施。农村生活垃圾有效处理率达到90%，县城生活垃圾无害化处理率达到100%。十是提高防灾减灾能力。建设梅州高新技术产业园区、东山教育基地、梅江河道芹洋半岛三处消防取水码头。实施500公里县乡公路安全生命防护工程。开展地质灾害详细调查，建立全市地质灾害信息系统。在山洪地质灾害易发区新建24个、升级改造44个自动气象站。

九、加强政府自身建设，不断提升服务水平

建设廉洁高效人民满意的政府，需要我们进一步改进作风、勇于担当、奋发有为。

坚持依法行政。提升运用法治思维和法治方式开展工作、推动发展的能力。落实重大行政决策法定程序，加强公众参与平台建设，健全重大行政决策终身责任追究制度及责任倒查机制。推行政府法律顾问制度，开展城乡建设与管理、环境保护、历史文化保护的相关立法工作。自觉接受市人大及其常委会的法律监督，支持市政协开展各项履职活动。认真办好人大代表议案、建议和政协委员提案，主动接受监督。加强行政复议和行政应诉工作，完善行政争议协调化解机制。推广“双随机一公开”监管工作，规范市场秩序。深化政务公开，完善网络问政平台，健全新闻发布及信息公开制度，打造政务微博品牌。强化公务员培训管理，提高队伍素质。

加强勤政廉政。坚持深入基层、深入群众，时刻关注民情，千方百计为群众排忧解难。推行政府部门服务大评比，广泛开展“群众最不满意事项”征集活动。全面落实权责清单和主体责任清单制度，从严规范权力运行。严格遵守党内政治生活准则和监督条例，加强和规范公务员政治生活。深入实践监督执纪“四种形态”，落实谈话提醒，构建抓早抓小工作机制，扎实推进惩治和预防腐败体系建设。强化审计监督，推动公共资金、国有资产、国有资源和领导干部经济责任履行情况审计全覆盖。进一步明晰政府与市场的边界，继续开展暖企行动，积极构建“亲”“清”新型政商关系。

营造主动作为、担当担责的氛围。紧盯目标，主动想办法、出实招，抓出实际效果。大力推行一线工作法和现场办公制，对重大项目、民生实事等重点工作，实行全过程、全方位督促检查。完善激励约束机制，落实“为官不为”问责暂行办法，坚决整治不作为、慢作为、乱作为等现象。认真贯彻落实“三个区分”原则和容错减责暂行办法，宽容干部在工作中特别是改革创新中的失误，充分调动干事创业积极性，打造一支敢于担当、善作善成的公务员队伍。

各位代表！实干创造未来，奋斗成就梦想！站在新的起点，让我们更加紧密地团结在以习近平同志为核心的党中央周围，在中央、省和市委的坚强领导下，抢抓机遇，开拓进取，加快建设“一区两带六组团”，为梅州全面建成小康社会而努力奋斗！
